# Structural analysis of DNA–protein complexes regulating the restriction–modification system *Esp*1396I

**DOI:** 10.1107/S174430911302126X

**Published:** 2013-08-19

**Authors:** Richard N. A. Martin, John E. McGeehan, Neil J. Ball, Simon D. Streeter, Sarah-Jane Thresh, G. G. Kneale

**Affiliations:** a Institute of Biomedical and Biomolecular Science, University of Portsmouth, King Henry I Street, Portsmouth, Hampshire PO1 2DY, England

**Keywords:** transcriptional regulation, DNA-binding proteins, helix–turn–helix motif, DNA distortion

## Abstract

The controller protein of the type II restriction–modification (RM) system *Esp*1396I binds to three distinct DNA operator sequences upstream of the methyltransferase and endonuclease genes in order to regulate their expression. Previous biophysical and crystallographic studies have shown molecular details of how the controller protein binds to the operator sites with very different affinities. Here, two protein–DNA co-crystal structures containing portions of unbound DNA from native operator sites are reported. The DNA in both complexes shows significant distortion in the region between the conserved symmetric sequences, similar to that of a DNA duplex when bound by the controller protein (C-protein), indicating that the naked DNA has an intrinsic tendency to bend when not bound to the C-protein. Moreover, the width of the major groove of the DNA adjacent to a bound C-protein dimer is observed to be significantly increased, supporting the idea that this DNA distortion contributes to the substantial cooperativity found when a second C-protein dimer binds to the operator to form the tetrameric repression complex.

## Abstract 2

Comparison of bound and unbound DNA in protein–DNA co-crystal complexes reveals insights into controller-protein binding and DNA distortion in transcriptional regulation.

## 1. Introduction

Bacterial restriction–modification (RM) systems act as a form of primitive immune system and prevent the establishment of foreign DNA (such as bacteriophages and plasmids) within bacteria (Wilson & Murray, 1991 ▶). It has been proposed that RM systems play a key role during the process of horizontal gene transfer between bacteria (Akiba *et al.*, 1960 ▶). An RM system is comprised of two complementary enzymes: a methyltransferase (M) to label ‘self’ DNA and an endonuclease (R) to cleave unlabelled (‘non-self’) DNA (Wilson & Murray, 1991 ▶). The plasmid-borne type II RM system *Esp*1396I has been well studied both *in vitro* and *in vivo* and reveals a temporal control mechanism that employs a controller protein (C-protein) encoded within the RM operon (Cesnaviciene *et al.*, 2003 ▶; Bogdanova *et al.*, 2008 ▶, 2009 ▶). This temporal control is necessary for the correct function of RM systems and to prevent auto-restriction (*i.e.* endo­nucleolytic cleavage of the bacterial chromosome and p*Esp*1396I plasmid).

The controller protein C.*Esp*1396I, and indeed all other C-proteins studied to date, have been shown to be homodimeric helix–turn–helix proteins that bind to pseudo-symmetrical DNA operator sequences (Ball *et al.*, 2009 ▶; McGeehan *et al.*, 2005 ▶; Streeter *et al.*, 2004 ▶; Kita *et al.*, 2002 ▶; Sawaya *et al.*, 2005 ▶). In C.*Esp*1396I and similar systems, it has been proposed that each DNA operator site comprises two ‘C-­boxes’ having pseudo-dyad symmetry with the consensus sequence GACT and a short spacer sequence in between them that is generally comprised of alternating purine–pryrimidine sequences (Streeter *et al.*, 2004 ▶; Knowle *et al.*, 2005 ▶; Sorokin *et al.*, 2009 ▶). Subsequently, it was found that the only specific contacts between C.*Esp*1396I and the C-boxes are to the GAC bases, so the C-box is perhaps better described as the trinucleotide GAC (and its symmetry-related sequence GTC) with the two C-boxes being separated by the spacer TATA, at least in the optimal binding site (Ball *et al.*, 2012 ▶). In addition, there are sequence-specific contacts to a conserved TG motif outside the C-boxes.

**Figure 1:**
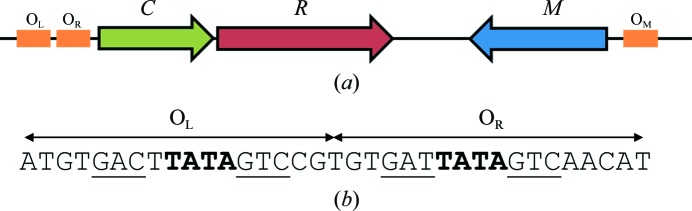
Organization of genes in the *Esp*1396I RM system. (*a*) The C-protein binding sites are coloured orange. The C-protein gene (*C*) is coloured green, the endonuclease gene (*R*) is coloured red and the methyltransferase gene (*M*) is coloured blue (adapted from Bogdanova *et al.*, 2009 ▶). (*b*) The O_L+R_ C-protein binding sites. The conserved GAC binding sites are underlined and the central TATA sequences are shown in bold. The TATA of the O_R_ binding site forms part of the ‘−35 box’ for the C/R genes

C.*Esp*1396I binds to three subtly different DNA sequences with vastly different affinities (*K*_d_ between 1 and 230 n*M*) that are located upstream of the C/R and M genes: O_M_ (which regulates the expression of the M gene), and O_L_ and O_R_ (which together control the expression of both the C and R genes) (Bogdanova *et al.*, 2009 ▶; Fig. 1 ▶). The X-­ray crystal structure of C.*Esp*1396I has been determined to high resolution as the free protein (Ball *et al.*, 2009 ▶) and as various protein–DNA complexes (McGeehan *et al.*, 2008 ▶, 2012 ▶; Ball *et al.*, 2012 ▶). All of the C-protein–DNA complex structures reveal distortion of the DNA helix owing to compression of the minor groove, which is either induced or stabilized by the bound C-protein. Owing to symmetry-related averaging of the tetrameric C-protein–DNA complex in the crystal structure (McGeehan *et al.*, 2008 ▶) further studies employed just single operator sites, to which a single C-­protein dimer bound (Ball *et al.*, 2012 ▶; McGeehan *et al.*, 2012 ▶). The O_L_ sequence yielded the highest resolution C-protein–DNA complex structure to date and showed the binding interface in great detail (McGeehan *et al.*, 2012 ▶). The subsequent O_M_ C-protein–DNA complex (Ball *et al.*, 2012 ▶) revealed conformational flexibility within the protein structure, enabling the protein to recognize different sequences but with quite different affinities. In contrast, the DNA was shown to have an almost identical structure in each case, with the overall bend angle being very similar to that of O_L_ and closely resembling that observed in the C/R tetrameric complex.

Here, we present two novel crystal structures that show the operator DNA structure corresponding to the O_R_ binding site, the lowest affinity of the three for C.*Esp*1396I. Each of these two structures, termed 19O_R_ and 25O_L_, are nucleoprotein complexes comprising a C-protein dimer and a DNA duplex. The 19O_R_ structure includes the entire O_R_ C-protein binding site. The 25O_L_ DNA sequence includes the O_L_ sequence plus half of the O_R_ C-protein binding site. The 25O_L_ complex allows the observation of part of the free (unbound) O_R_ sequence, unlike the previously published 35O_L+R_ complex that has the complete O_R_ sequence. In the latter complex, owing to the high cooperativity between sites, the C-protein forms a tetramer (*i.e.* two dimers) on the 35O_L+R_ DNA (McGeehan *et al.*, 2008 ▶).

## 2. Materials and methods

### 2.1. Crystallization

Expression and purification of native C.*Esp*1396I was carried out as described previously (McGeehan *et al.*, 2008 ▶). In brief, C.*Esp*1396I was overexpressed in *Escherichia coli* strain BL21 (DE3) pLysS using the pET-28b vector to introduce an N-terminal six-histidine sequence. C.*Esp*1396I was purified using nickel-affinity chromatography and size-exclusion chromatography. Prior to the crystallization trials, the six-histidine tag was removed using thrombin. The DNA oligo­nucleotides for crystallization of the 19O_R_ complex (5′-TGTGT­GATTATAGTCAACA-3′ and its complementary strand) and the 25O_L_ complex (5′-ATGTGACTTATAGTCGTGTGATTA-3′ and its complementary strand) were synthesized by ATDBio and Eurogentec, respectively, and were purified by RP-HPLC. The complementary oligonucleotides were annealed by heating to 353 K followed by cooling and the duplexes were further purified using gel electrophoresis. Initial cocrystallization was carried out using a HoneyBee X8 crystallization robot (Cronus Technologies) and sparse-matrix screening using the PACT Premier and JCSG+ screens (Molecular Dimensions Ltd) at varying molar ratios of C.*Esp*1396I to DNA duplex. Crystals of the 19O_R_ complex formed by vapour diffusion in 0.1 *M* propionic acid, sodium cacodylate and bis-tris propane (PCB) buffer pH 4.0 with 25%(*w*/*v*) PEG 3350 at a molar protein:DNA ratio of 1:1. However, these crystals were of insufficient size for diffraction experiments, so a microseeding approach was employed (D’Arcy *et al.*, 2007 ▶). This produced much larger crystals in 0.1 *M* PCB buffer pH 5.0, 25%(*w*/*v*) PEG 3350, 10 m*M* spermidine. The crystals were confirmed to contain both protein and DNA by washing them and subsequently dissolving them in dH_2_O before taking a UV absorbance spectrum. Crystals of the 25O_L_ complex formed in 0.1 *M* PCB buffer pH 4.0, 20%(*w*/*v*) PEG 1500, 10 m*M* spermidine at a molar protein:DNA ratio of 2:1.

### 2.2. X-ray diffraction data collection and refinement

The 19O_R_ and 25O_L_ crystals were transferred to a cryoprotectant containing 25%(*v*/*v*) glycerol or 20%(*v*/*v*) PEG 400, respectively, and flash-cooled in liquid nitrogen. For the 19O_R_ crystal, 180 images of 1° oscillation were collected on beamline I02 at the Diamond Light Source (DLS), Oxfordshire at a wavelength of 0.98 Å using an ADSC Quantum 315r CCD detector at 100 K. For the 25O_L_ crystal, 120 images of 1° oscillation were collected using an ADSC Q4R CCD detector at 100 K on beamline ID14-4 at the ESRF, Grenoble.

The data were processed using either *MOSFLM* (Leslie, 1992 ▶) and *AIMLESS* (Winn *et al.*, 2011 ▶; Evans, 2006 ▶, 2011 ▶) or *XDS* and *XSCALE* (Kabsch, 2010 ▶) and a molecular-replacement solution was found by *Phaser* (McCoy *et al.*, 2007 ▶) using the native free protein structure as a search model (Ball *et al.*, 2009 ▶; PDB entry 3g5g). The DNA was built by hand in *Coot* (Emsley & Cowtan, 2004 ▶) and was subsequently refined using *REFMAC*5 (Murshudov *et al.*, 2011 ▶) and *phenix.refine* (Afonine *et al.*, 2005 ▶). Data-processing and refinement statistics are summarized in Table 1 ▶. The completed models were deposited in the PDB with accession codes 4i8t (19O_R_) and 4iwr (25O_L_).

## 3. Results

### 3.1. X-ray diffraction and structure solution

**Figure 2:**
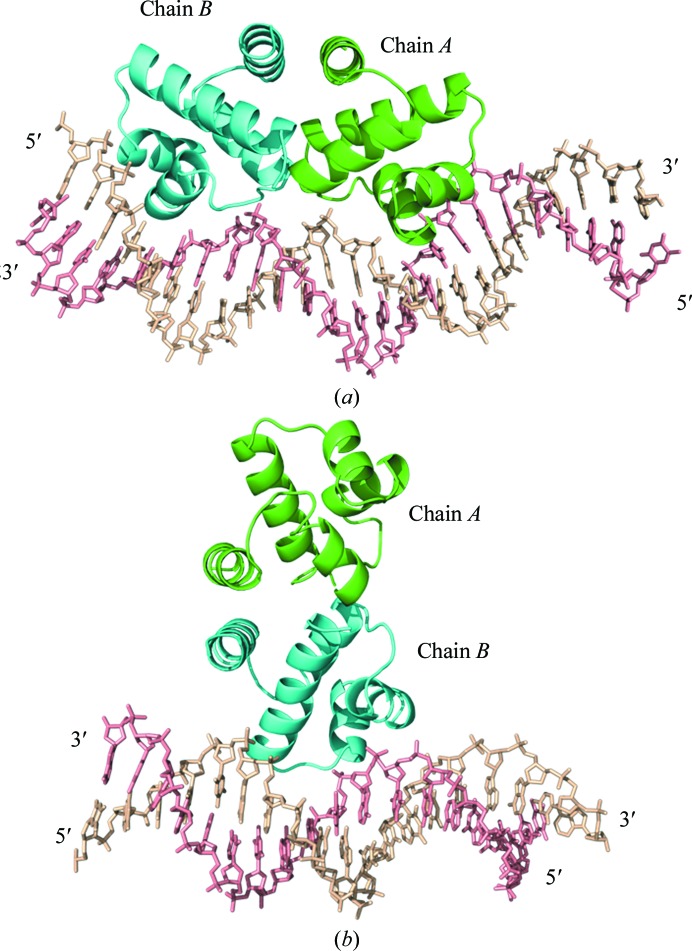
C-protein–DNA complexes. (*a*) C.*Esp*1396I dimer bound to a 25 bp DNA duplex containing the native operator O_L_ and half of the O_R_ sequence (PDB entry 4iwr). (*b*) C.*Esp*1369I dimer interacting with a 19 bp DNA duplex containing the native operator O_R_ (PDB entry 4i8t).

**Figure 3:**
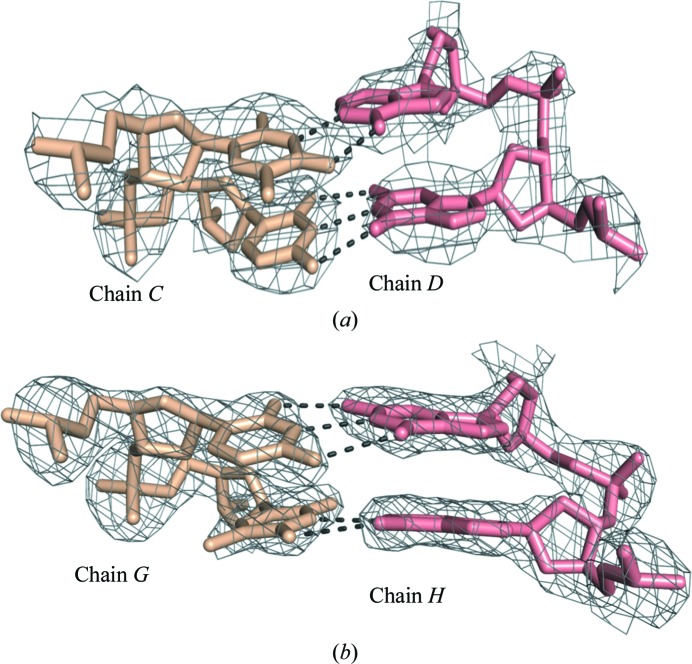
Representative 2*F*_o_ − *F*_c_ electron-density maps. (*a*) Base pairs of T14 and C15 of chain *C* with G6 and A7 of chain *D* from the 19O_R_ DNA. (*b*) Base pairs between chain *G* (C7 and T8) and chain *H* (A18 and G19) from the 25O_L_ DNA. Hydrogen bonds are shown as dashed lines. 2*F*_o_ − *F*_c_ electron-density maps are contoured at 0.16 and 0.32 e Å^−3^ for 19O_R_ and 25O_L_, respectively. The images were generated using *PyMOL*.

The 19O_R_ crystals showed weak isotropic diffraction extending to 3 Å resolution. The scaling program *AIMLESS* (Evans, 2006 ▶, 2011 ▶; Winn *et al.*, 2011 ▶) gave a high *R*_merge_ for the outer shell, but inspection of the electron-density maps and use of the CC_1/2_ metric (Karplus & Diederichs, 2012 ▶) gave a clear indication that the data were acceptable to 3 Å resolution, with a final *R*_work_ and *R*_free_ of 0.28 and 0.36 for the outer shell. The structure was refined in space group *C*2 with one copy of the complex per asymmetric unit (Fig. 2 ▶). The resulting 2*F*_o_ − *F*_c_ maps were of good quality for the resolution (Fig. 3 ▶).

**Figure 4:**
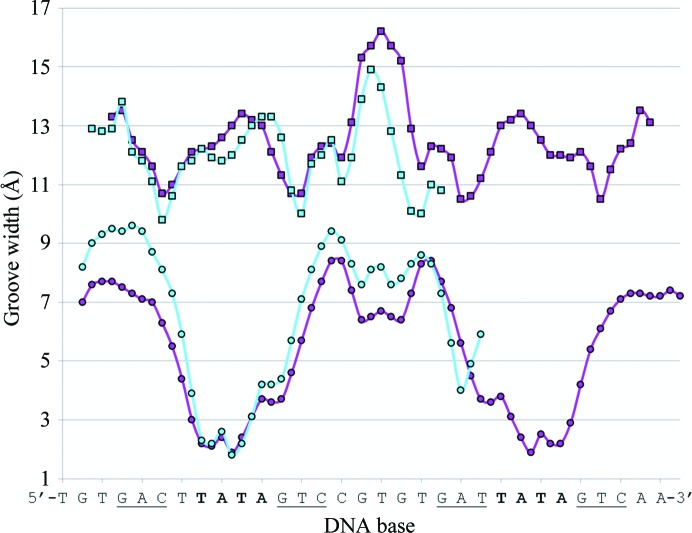
DNA groove-width analysis of the 25O_L_ DNA. Groove-width analysis of the 25O_L_ DNA (cyan) compared with the published 35O_L+R_ complex (PDB entry 3clc; magenta; McGeehan *et al.*, 2008 ▶). Upper curve, major groove; lower curve, minor groove. The DNA sequence of the 25O_L_ sequence is shown below. The TATA sequences are shown in bold and the DNA recognition bases are underlined.

The best crystals of the 25O_L_ complex diffracted to ∼2.3 Å resolution. The structure was refined in space group *P*3_2_ with two copies of the complex per asymmetric unit. The DNA was easily modelled into the electron density for the section that was bound to C.*Esp*1396I (McGeehan *et al.*, 2012 ▶). However, owing to the high degree of flexibility of the additional six base pairs, these were more difficult to model and were primarily based on the positions of the backbone phosphate groups since these gave much higher peaks in the electron density relative to the bases. This flexibility resulted in *B* factors of approximately 130 Å^2^ in this unbound section of the DNA compared with an average *B* factor of approximately 15 Å^2^ in the protein-bound portion of the DNA (Supplementary Fig. S1^1^). DNA groove-width analysis (Fig. 4 ▶) was performed using the *Curves* ^+^ server (Lavery *et al.*, 2009 ▶).

### 3.2. The 19O_R_ structure

The overall fold of C.*Esp*1396I in the 19O_R_ structure closely matches that of the free protein structure (PDB entry 3g5g; Ball *et al.*, 2009 ▶), with an overall r.m.s.d. of 0.65 Å over all observable main-chain atoms. The flexible loop regions are found in the major loop conformation observed in the free protein structure (Ball *et al.*, 2009 ▶). However, owing to the limited resolution, not all side chains could be placed with high confidence other than those that are highly ordered and binding to symmetry-related protein chains or to the DNA.

Surprisingly, the protein dimer does not bind to the DNA in the usual manner *via* the helix–turn–helix (HTH) motif; instead, it binds ‘end-on’ to the DNA helix, resulting in very few protein–DNA interactions (Fig. 2 ▶ *b*). This non-biological complex reflects the low intrinsic binding affinity at a single O_R_ site. It is only when a C-protein dimer is bound to the adjacent O_L_ site that the protein binds in the expected manner (as observed in the complex with the 35 bp O_L_ + O_R_ operator DNA). This arises from the high degree of cooperativity that increases the affinity for the O_R_ site by two orders of magnitude when a C-protein dimer is bound at the O_L_ site.

In the 19O_R_ crystal structure, each protein dimer contacts four DNA duplexes and two protein subunits belonging to adjacent asymmetric units. The protein–protein contacts involve two tyrosines (Tyr29 from each subunit) stacking against each other in a manner similar to that previously observed, but with the addition of hydrogen bonds between Tyr29 and Glu25 and Asp26 (Ball *et al.*, 2009 ▶, 2012 ▶; McGeehan *et al.*, 2012 ▶). The only clear contacts between the C-­protein and the DNA occur between the protein side chains and the phosphate groups in the DNA backbone.

The overall conformation of the DNA duplex in the 19O_R_ structure does not conform to the canonical B-form; it is significantly distorted and resembles the biologically bound conformation previously observed in the 19O_L_ structure (Figs. 4 ▶ and 5 ▶). The overall bend of 42° is a little less than that observed in the biologically bound O_L_ complex (54°), but the DNA retains the reduced minor groove in the central spacer between the two C-boxes, despite the lack of significant interactions with the HTH motif. The bend in the DNA is centred at the TATA sequence between the C-boxes (Fig. 1 ▶), as observed in other C-protein complexes. The bent DNA structure that we observe here is most likely to reflect a natural propensity to bend at this sequence, and in biologically relevant complexes is enhanced and stabilized by interactions with the HTH motif, as observed in the tetrameric complex and in the higher affinity O_L_ and O_M_ complexes (McGeehan *et al.*, 2008 ▶, 2012 ▶; Ball *et al.*, 2012 ▶).

### 3.3. The 25O_L_ structure

**Figure 5:**
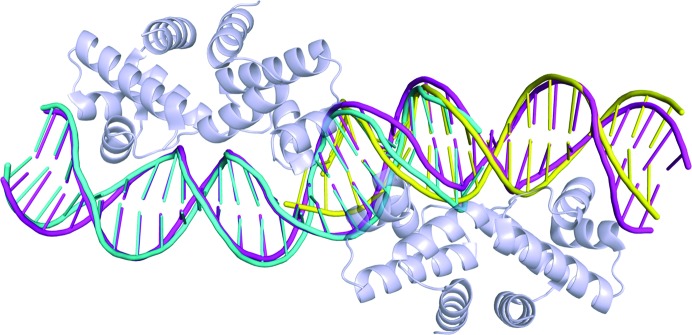
DNA structural comparisons. The 25O_L_ DNA (cyan) and the 19O_R_ DNA (yellow) are aligned against the 35O_L+R_ DNA (magenta). The protein dimers in the latter complex are displayed in grey.

There were no significant differences between the conformations of the two complexes in the asymmetric unit. The 25O_L_ protein structure (Fig. 2 ▶ *a*) closely resembles that of the previously published 19O_L_ protein–DNA complex structure, with an overall r.m.s.d. of 0.48 Å for the main-chain atoms of the protein monomers and 0.92 Å for the corresponding 18 bp of the DNA (Fig. 5 ▶). The same specific and nonspecific protein–DNA contacts were visible in the structure. However, owing to the longer DNA component of the complex, the crystal-packing interactions between the proteins are markedly different.

The only observable protein–protein contacts between crystallo­graphic symmetry-related dimers again involve the stacking of Tyr29 side chains, together with a hydrogen bond between Tyr29 and Asp26 of the symmetry-related subunit. There are very few protein–DNA interactions between chains that are not within the biological complex and all involve interactions between protein side chains and phosphate groups on the DNA backbone. The crystallographic DNA–DNA interactions between symmetry-related molecules are limited to stacking between the terminal base pair A1–T25 (chains *C* and *D*) and the corresponding A–T base pair of chains *G* and *H*. This causes the DNA to form a pseudo-continuous double helix.

The width of the major groove in the 25O_L_ DNA varies from 10 to 15 Å in a sequence-dependent manner (Fig. 4 ▶). Likewise, the minor-groove width varies from 2 to 9 Å. The portion of the 25O_L_ structure that contains the first C-box (O_L_) overlays very closely with the relevant sequence in the 35O_L+R_ tetramer structure, with an r.m.s.d. of 0.92 Å (Fig. 4 ▶). The remainder of the DNA that is not bound by the protein also follows a similar path to that of the DNA in the tetrameric complex. It is noteworthy that the major groove that is significantly widened in the centre of the tetrameric 35 bp complex is also widened in the equivalent region of the 25O_L_ complex, even though this region of the DNA is unbound (Figs. 4 ▶ and 5 ▶).

## 4. Discussion

These novel protein–DNA complexes enable comparison of the conformation of the DNA sequence before and after C-protein binding. C-proteins, in common with many helix–turn–helix DNA-binding proteins, bend and distort their DNA-binding sites in order to access the bases for sequence recognition (Kita *et al.*, 2002 ▶; Papapanagiotou *et al.*, 2007 ▶; McGeehan *et al.*, 2008 ▶, 2012 ▶; Ball *et al.*, 2012 ▶). The 19O_R_ structure presented here shows that even in the absence of specific protein–DNA contacts the DNA sequence at the O_R_ operator is compressed at the minor groove, greatly reducing the energy penalty of DNA distortion following C-protein binding. Using circular dichroism, it has been shown that significant structural deformation of the DNA occurs when the controller protein C.*Ahd*I binds its operator sequence in solution (Papapanagiotou *et al.*, 2007 ▶). Presumably, the same will apply to the O_L_ and O_M_ operators of the *Esp*1396I RM system, which all contain the central TATA sequence.

The observed path of the DNA within the 25O_L_ complex supports the proposal that the binding of the first C-protein to the O_L_ site assists in opening up the major groove of the O_R_ site in preparation for binding the second C-protein dimer, thus compensating for the weaker intrinsic binding affinity of the O_R_ site. This provides a significant component of the observed cooperativity of binding between the two adjacent operator sites, in addition to specific protein–protein contacts between adjacent dimers (McGeehan *et al.*, 2008 ▶). A similar mechanism based on DNA distortion has been proposed for the cooperative binding of the QacR transcriptional regulator to its operator site (Schumacher *et al.*, 2002 ▶), but in this case there were no additional protein–protein interactions contributing to the cooperativity. The downstream effects of binding one protein dimer on the structure of the adjacent DNA, thereby enhancing its DNA-binding affinity for a second protein dimer, could represent a more general mechanism of transcriptional control.

## Supplementary Material

PDB reference: C.*Esp*1396I, complex with 19 bp DNA duplex, 4i8t

PDB reference: complex with 25 bp DNA duplex, 4iwr

Supplementary material file. DOI: 10.1107/S174430911302126X/kw5075sup1.pdf

## Figures and Tables

**Table 1:** X-ray crystal data, refinement and model statistics Values in parentheses are for the highest resolution shell.

Complex	19O_R_	25O_L_
PDB code	4i8t	4iwr
Space group	*C*2	*P*3_2_
Unit-cell parameters (Å, °)	*a* = 75.51, *b* = 60.86, *c* = 80.35, α = γ = 90, β = 113.47	*a* = *b* = 48.02, *c* = 218.35, α = β = 90, γ = 120
Solvent content (%)	50	44
Complexes in asymmetric unit	1	2
R.m.s. distance between complexes (Å)	N/A	0.25
Data collection		
Beamline	I02, DLS	ID14-4, ESRF
Detector	ADSC Q315r	ADSC Q4R
Wavelength (Å)	0.979	0.933
Resolution (Å)	3.0	2.4
No. of measured reflections	22560	56668
No. of unique reflections	6809	11658
Completeness (%)	99.9 (100)	97.8 (94.6)
〈*I*/σ(*I*)〉	5.9 (0.9)	10.2 (2.0)
Multiplicity	3.3 (3.3)	4.9 (4.3)
*R*_merge_ †	0.158 (1.07)	0.048 (0.542)
CC_1/2_ ‡	0.988 (0.652)	N/A
Wilson *B* factor (Å^2^)	58	59
Refinement parameters		
*R*_work_/*R*_free_	0.235/0.300	0.197/0.259
No. of atoms		
Protein	1253	2463
DNA	776	2050
Average *B* factor (Å^2^)		
Protein	83	14
DNA	93	30
R.m.s. deviations from ideal geometry§		
Bond lengths (Å)	0.002	0.011
Bond angles (°)	0.675	1.52
Ramachandran outliers (%)	3.6	2.7
*MolProbity* ¶ score	2.8	2.5
Clashscore	11	6

† *R*_merge_ = 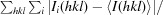 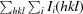, where 〈*I*(*hkl*)〉 is the average of Friedel-related observations of a unique reflection.

‡ CC* = [2CC_1/2_/(1 + CC_1/2_)]^1/2^, where CC* is as estimate of CC_true_ based on a finite sample size.

§ Engh & Huber (2001 ▶).

¶ Chen *et al.* (2010 ▶).

## Footnotes

1 Supplementary material has been deposited in the IUCr electronic archive (Reference: KW5075).
