# Supplementary material for: Structural analysis of DNA–protein complexes regulating the restriction–modification system Esp1396I
Source: Acta Crystallogr Sect F Struct Biol Cryst Commun. 2013 Aug 19;69(Pt 9):962–6. doi: 10.1107/S174430911302126X (PMC3758141; doi:10.1107/S174430911302126X)
Supplement: Supplementary file 1 [file f-69-00962-sup1.pdf]

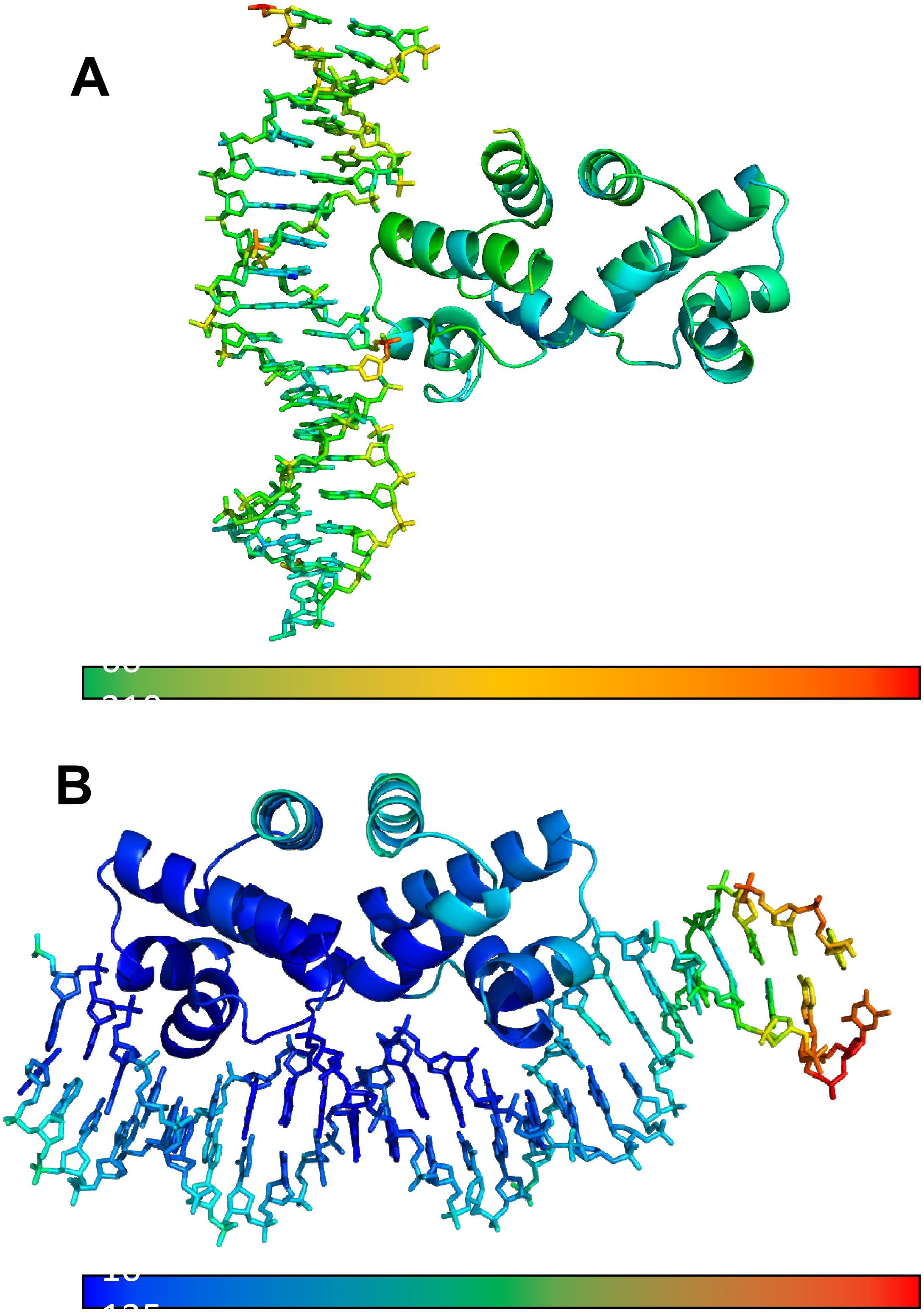

Figure S1: B-Factors of the $19O_R$ and $25O_L$ structures. A: $19O_R$ and **B**: $25O_L$ structures coloured by B-factor (scale bars respective to the structure above).
